# QoS-Aware Joint Task Scheduling and Resource Allocation in Vehicular Edge Computing

**DOI:** 10.3390/s22239340

**Published:** 2022-11-30

**Authors:** Chenhong Cao, Meijia Su, Shengyu Duan, Miaoling Dai, Jiangtao Li, Yufeng Li

**Affiliations:** 1 School of Computer Engineering and Science, Shanghai University, Shanghai 200444, China; 2 Purple Mountain Laboratories, Nanjing 211111, China

**Keywords:** vehicular edge computing, resource allocation, computation offloading, multi-objective optimization

## Abstract

Vehicular edge computing (VEC) has emerged in the Internet of Vehicles (IoV) as a new paradigm that offloads computation tasks to Road Side Units (RSU), aiming to thereby reduce the processing delay and resource consumption of vehicles. Ideal computation offloading policies for VEC are expected to achieve both low latency and low energy consumption. Although existing works have made great contributions, they rarely consider the coordination of multiple RSUs and the individual Quality of Service (QoS) requirements of different applications, resulting in suboptimal offloading policies. In this paper we present FEVEC, a Fast and Energy-efficient VEC framework, with the objective of realizing an optimal offloading strategy that minimizes both delay and energy consumption. FEVEC coordinates multiple RSUs and considers the application-specific QoS requirements. We formalize the computation offloading problem as a multi-objective optimization problem by jointly optimizing offloading decisions and resource allocation, which is a mixed-integer nonlinear programming (MINLP) problem and NP-hard. We propose MOV, a Multi-Objective computing offloading method for VEC. First, vehicle prejudgment is proposed to meet the requirements of different applications by considering the maximum tolerance delay related to the current vehicle speed. Second, an improved Non-dominated Sorting Genetic Algorithm-II (NSGA-II) is adopted to obtain the Pareto-optimal solutions with low complexity. Finally, the optimal offloading strategy is selected for QoS maximization. Extensive evaluation results based on real and simulated vehicle trajectories verify that the average QoS value of MOV is improved by 20% compared with the state-of-the-art VEC mechanism.

## 1. Introduction

With the rapid development of sensing and communication technologies in the automotive industry, the IoV has become a fundamental information infrastructure for intelligent transportation systems [1]. IoV technology facilitates a broad range of compelling applications, including traffic management, navigation, and passenger entertainment [2]. These applications are usually computation-intensive, and have stringent timelines [3]. For example, connected vehicles need to process an enormous amount of data in real time (at often GB/s rates) with extremely tight latency and energy cost constraints [4]. Although cloud computing can provide powerful computing resources, long-distance data transmission and heavy transmission overhead may cause unacceptable latency and affect the application’s QoS [5].

Recent advances in VEC [6] create new opportunities for vehicular applications processing in a timely manner by integrating MEC (Mobile Edge Computing) into vehicular networks. VEC extends centralized computing capability to the network edge which is in proximity to the vehicle [2]. Specifically, VEC can reduce the end-to-end delay and save both the communication and computational resource of the vehicle by offloading computation tasks to an RSU equipped with roadside edge servers (RES) [3]. Unlike traditional MEC, VEC faces more dynamic network conditions due to the fast mobility of vehicles, and it has a more rigorous timeliness requirement in the driving scenario. Although VEC offers significant benefits, it is critically reliant on the complicated communication characteristics between vehicles and RSUs and the computational resources they provide. A key challenge faced by VEC is the development of appropriate offloading strategies that deal with frequently changing communication conditions and limited computational resources [2,6].

Recently, there has been growing interest in studies related to the ideal computation offloading strategy via vehicle-to-infrastructure (V2I) and vehicle-to-vehicle (V2V) communications, with the expectation of achieving both low latency and low energy consumption. Researchers have utilized powerful vehicles to assist in offloading via V2V; however, this approach cannot provide stable service due to unreliable V2V links and dynamic computation capacity [7]. To improve reliability, many works have made extensive innovations to offload tasks to the RSU via V2I [8,9]. However, these often neglect highly dynamic network conditions or limited edge resources, meaning that the user may suffer from service disruption when the vehicle travels out of the current RSU, and do not consider cooperation between adjacent RSUs. Recent research has promoted the use of reinforcement learning (RL) to cope with network and resource dynamics. However, RL-based solutions are limited to a small number of vehicles (e.g., the maximum number of vehicles in the evaluation of [7] is 25) due to the curse-of-dimensionality problem.

Further, existing works often neglect application-specific QoS requirements, which can lead to sub-optimal offloading policies. Note that different tasks usually have different requirements in VEC. For example, a navigation task has higher delay demand than energy consumption, while an entertainment task may prefer low energy consumption to low latency. Moreover, different vehicles have different speeds over time, which has an impact on the maximum delay tolerance with respect to a given task.

Motivated by these limitations, we present FEVEC, a Fast and Energy-efficient VEC framework which considers the application-specific QoS requirements and makes optimal offloading decisions, thereby minimizing both delay and energy consumption by coordinating multiple RSUs within the coverage area of RSU. FEVEC estimates the uplink rate of each vehicle according to its distance to the RSU and the number of vehicles in the RSU coverage. To estimate the energy consumption, FEVEC establishes a resource consumption model to analyze the computing and transmission energy consumption. Moreover, FEVEC considers the individual needs of different applications in its offloading policy as well as the relationship between current speed and maximum tolerance delay. Finally, the offloading decisions are made in terms of the allocation of communication resources and computing resources to support various types of services. The main contributions of this work are summarized as follows:

1. We propose FEVEC, a Fast and Energy-efficient VEC framework to find the optimal offloading strategy. FEVEC comprehensively considers frequently changing network conditions and limited computation resources, aiming to minimize overall delay and energy consumption.
2. We formalize the problem of devising an offloading strategy as a multi-objective optimization problem, and propose a multi-objective computing offloading method for VEC named MOV to obtain the optimal offloading policy. Compared with other works, this approach considers the collaboration between multiple RSUs and the application-specific QoS requirement, where an improved Non-dominated Sorting Genetic Algorithm-II (NSGA-II) is employed to generate the Pareto-optimal solutions with low complexity.
3. We evaluate FEVEC using real-world and simulated vehicle trajectories. Extensive evaluations are provided to demonstrate the effectiveness of our proposed MOV compared to the state-of-the-art schemes; the proposed method leads to an improvement of about 20% on average compared with PSOCO [3].

The rest of this paper is organized as follows. Section 2 describes the motivation behind this paper. Section 3 introduces the VEC offloading framework and formalizes it as a multi-objective optimization problem for an urban IoV scenario. Section 4 provides a multi-objective computing offloading method for VEC (MOV) to solve the problem with low complexity. Section 5 describes the evaluation results of the Pareto-optimal solutions and the overall QoS performance. Section 6 discusses related works involving different offloading strategies. Finally, Section 7 concludes this paper and proposes future research directions.

## 2. Motivation Example

To illustrate the motivation behind FEVEC, we provide two examples where existing techniques can only make sub-optimal offloading decisions. We compare FEVEC to PSOCO [3], a state-of-the-art VEC offloading mechanism which aims to minimize delay and energy cost. PSOCO considers offloading the computation tasks to the nearest RSU, ignoring the possibility of collaboration between multiple RSUs. Here, we assume that two RSUs (RSU1 and RSU2) are adjacent and the coverage radius of each RSU is 200 m. Details are provided in Table 1.

**Figure 1:**
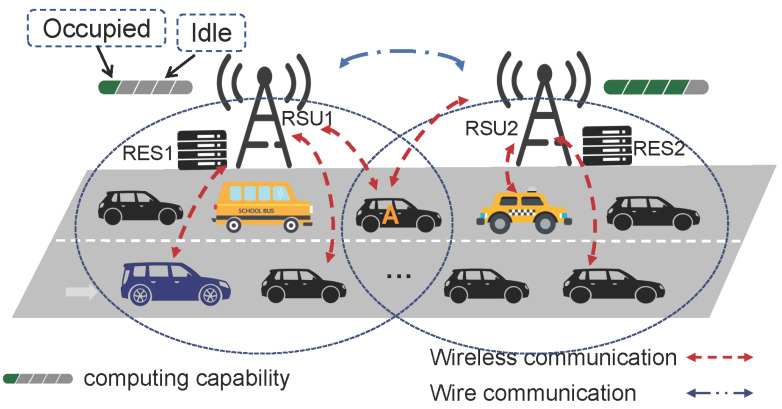
FEVEC framework.

Example 1: The first example considers a simple scenario where only one vehicle is in the communication range of two RSUs. As shown in Figure 1, suppose that only vehicle *A* is traveling along a road, leaving RSU1 and approaching RSU2. At this moment, vehicle *A* is 160 m away from RSU1 and 190 m away from RSU2. Vehicle *A* generates a task with a data size of 5 MB. PSOCO makes the offloading decision to offload part of the data (80%) to RSU1 and execute the other part locally. The corresponding delay and energy consumption can be calculated as 0.767 s and 4.43 J. As a comparison, FEVEC can coordinate two RSUs and make better offloading decisions, achieving 0.507 s delay and 1.38 J energy consumption.

Example 2: In our second example, vehicles run multiple applications with different QoS requirements. Consider a scenario in which a vehicle is performing a navigation task with highly delay requirement, while at the same time another vehicle is running an entertainment task that prefers low energy consumption to low latency. Because PSOCO considers the latency and energy costs to be equally important for all applications, PSOCO makes sub-optimal offloading decisions for both tasks, achieving 2 s latency and 3.27 J energy consumption. In contrast, FEVEC is able to consider the individual requirements of different applications and make more reasonable offloading decisions. Therefore, FEVEC can achieve 1.45 s latency and 2.76 J energy consumption, reaching a QoS value above 0.9.

In summary, a number of difficulties exist in the PSOCO mechanism, including inability to (1) coordinate multiple RSUs and (2) consider the individual QoS requirements of different applications. These difficulties can hinder PSOCO from making optimal offloading decisions in a practical scenario. This motivates us to design an approach that can make optimal offloading decisions by carefully considering the cooperation of multiple RSUs as well as the application-specific QoS requirements. Thus, we present FEVEC, which is able to cooperate with multiple RSUs within their communication range while considering frequently changing network condition and limited computation resources. In addition, our offloading mechanism is able to satisfy the varying QoS requirements of different applications.

## 3. System Model and Problem Formulation

In this section, we introduce the overall system model and problem formulation. We first introduce the definition and assumption, then describe the system model, including the communication and computation model. Finally, the problem of multi-objective optimization is formalized, which is NP-hard [11]. Key notations are described in Table 2.

### 3.1. Definitions and Assumptions

**VEC framework design:** We consider a practical urban IoV scenario for FEVEC consisting of a unidirectional road along which the coverage of RSUs overlaps, as shown in Figure 1. In FEVEC, RSUs are located along the roadside; the communication radius of each RSU is *r*, meaning that a vehicle can be covered by up to two RSUs. Each RSU is equipped with one RES with limited computing capability, which is capable of dealing with complex computation tasks in parallel through reasonable resource allocation. An RSU can communicate with another RSU via wire link to share information and further make the optimal offloading decisions. We assume that there are *N* vehicles within the coverage of two adjacent RSUs, and each vehicle is equipped with a single antenna that can be connected to one RSU through V2I wireless communication to broadcast status messages.

**Time-slot based system:** We consider a system based on time slots in which both the number and location of vehicles change dynamically over time, along with their communication and computation conditions. Without loss of generality, we suppose that the offloading task at any slot can be completed before the next slot within the coverage area of two adjacent RSUs.

**Task definition:** At time slot *t*, various tasks are generated from vehicles for road safety and passenger entertainment. We denote a computation task of vehicle *n* as $T_{n}$, $T_{n}=\left\{D_{n},C_{n},t_{max},\lambda_{n},\mu_{n}\right\}$. Here, $D_{n}$ is the data size of the task $T_{n}$ on the vehicle *n*, $C_{n}$ is the computation intensity (in CPU cycles per bit), and $t_{max}$ is the delay tolerance of task $T_{n}$, which is related the current speed; $\lambda_{n}$ and $\mu_{n}$ are weight coefficients, indicating the requirements for delay and energy consumption, respectively. The computation task is divided into several parts, and has up to three offloading destinations, namely, local, RSU1, and RSU2, which are processed in parallel. We define $\alpha_{n}$, $\beta_{n}$, and $\gamma_{n}$ ($0\leq \alpha_{n},\beta_{n},\gamma_{n}\leq 1$) as the offloading decision variables of task $T_{n}$, which denote the offloading ratio of a task to RSU1, RSU2, and local, respectively. Thus, the amount of data offloaded to RSU1 and RSU2 is $\alpha_{n}D_{n}$ bits and $\beta_{n}D_{n}$ bits, and the amount of data executed locally is $\gamma_{n}D_{n}$ bits.

We assume that a vehicle *n* runs at a speed *s*, and that the distance between vehicle *n* and an RSU is $d_{n}^{RSU}$. Due to the high-speed mobility of the vehicle, the vehicle *n* might leave the communication range of the current RSU during task transmission. We denote the dwell time of vehicle *n* within the coverage of the current RSU as $t_{stay}$, which can be calculated as

(1) $$t_{\mathrm{stay}\;}=\frac{l}{s},$$

where *l* is the distance between vehicle *n* and the coverage edge of the current RSU in the direction of the vehicle. Both *s* and $d_{n}^{RSU}$ can be known from GPS data [12]. For example, if vehicle *n* is traveling in the direction of the RSU, we have $l=r+d_{n}^{RSU}$. Otherwise, if vehicle *n* is moving away from the current RSU, *l* is calculated as $l=r-d_{n}^{RSU}$.

**Offloading procedure:** For an offloading task of (1-$\gamma_{n}$)$D_{n}$ bits, the procedure includes three parts. First, the task is uploaded to the RSU, then processed, and finally the RSU returns the result to the vehicle. Specifically, when the vehicle leaves RSU1 and approaches RSU2, it can adaptively upload $\alpha_{n}D_{n}$-bit (which can be 0) data to RSU1 according to network conditions. After driving into RSU2, the result of RSU1 is transmitted to RSU2 and finally returned to the vehicle when RSU2 finishes. For example, in vehicle video monitoring, a task defined as processing the video stream generated by each time slot, the offloading ratio is the number of frames that are offloaded to the edge for execution.

### 3.2. Communication Model

For V2I communication, the non-orthogonal multiple access (NOMA) technique is leveraged to provide massive connectivity. The RES transmits signals to multiple vehicles and separates the bandwidth to multiple subchannels. The vehicle can use multiple subchannels for transmission, and a subchannel can be accessed by multiple vehicles [13]. This technology is different from traditional wireless communication in IoT, where WiFi and Bluetooth are more unpredictable.

Based on the transmission task, we model the channel between RSU and the vehicle during one slot by considering the distance between them and the number of vehicles in RSU coverage to estimate the link rate. Accordingly, the available uplink data rate of vehicle *n* over subchannel *m* in RSU1 after successive interference cancellation (SIC) [13], $r_{n,m}^{RSU1,up}$, is

(2) $$r_{n,m}^{RSU1,up}=\frac{W_{1}}{M}\log_{2}\left(1+\frac{p_{n,m}^{RSU1,up}h_{n,m}^{RSU1,up}}{\xi_{n,m}^{RSU1,up}+\sigma^{m}{\left(d_{n}^{RSU1}\right)}^{v}}\right),$$

where we define the uplink bandwidth of RSU1 as $W_{1}$, *M* is the number of total subchannels of the RSU1 server, $p_{n,m}^{RSU1,up}$ is the transmission power, $h_{n,m}^{RSU1,up}$ is the power gain, $\xi_{n,m}^{RSU1,up}$ and $\sigma^{m}$ denote the interference signal power from other vehicles on channel *m* and the White Gaussian noise power, respectively, $d_{n}^{RSU1}$ is the distance between vehicle *n* and RSU1, and *v* is the path loss exponent. Now, let $a_{n,m}^{RSU1,up}$ indicate the uplink binary channel allocation indicator, while $a_{n,m}^{RSU1,up}=1$, expressing subchannel *m*, is allocated to vehicle *n* on the uplink of RSU1; otherwise, $a_{n,m}^{RSU1,up}=0$. Therefore, the data transmission rate of vehicle *n* on the uplink of RSU1 is

(3) $$R_{n}^{RSU1,up}=\sum_{m=1}^{M}a_{n,m}^{RSU1,up}r_{n,m}^{RSU1,up}.$$

Thus, the upload time of $\alpha_{n}D_{n}$ bits to RSU1 is $t_{n}^{RSU1,up}$,

(4) $$t_{n}^{RSU1,up}=\frac{\alpha_{n}D_{n}}{R_{n}^{RSU1,up}}.$$

Accordingly, the energy consumption of uploading the $\alpha_{n}D_{n}$ bits of data to RSU1 is $E_{n}^{RSU1,up}$,

(5) $$E_{n}^{RSU1,up}=\sum_{m=1}^{M}p_{n,m}^{RSU1,up}t_{n}^{RSU1,up}.$$

Because the delay and energy consumption for the downlink is much less than for the uplink, for the sake of simplicity we ignore the delay and energy consumption in the downlink [2].

### 3.3. Computation Model

Further, we formulate a task computing model of the vehicle and RSU in which the resource allocation of different platforms is considered. We define the maximum computing capability of a vehicle *n* as $F_{n}$ (CPU cycles/s), while $f_{n}^{l}\left(0\leq f_{n}^{l}\leq F_{n}\right)$ represents the computing resources allocated for task $T_{n}$ when $\gamma_{n}D_{n}$ bits of data are executed locally. Then, the time required for local task execution, $t_{n}^{l}$, can be calculated by

(6) $$t_{n}^{l}=\frac{C_{n}\gamma_{n}D_{n}}{f_{n}^{l}}.$$

The energy consumption for $\gamma_{n}D_{n}$ bits of data by vehicle *n* is

(7) $$E_{n}^{l}=t_{n}^{l}p_{n}^{l}=k_{n}C_{n}\gamma_{n}D_{n}{\left(f_{n}^{l}\right)}^{2},$$

where $p_{n}^{l}$ is the power consumption of vehicle *n*, $p_{n}^{l}=k_{n}{\left(f_{n}^{l}\right)}^{3}$, and $k_{n}$ is a chip coefficient related to the power available to vehicle *n* [3].

For a computation task with $\alpha_{n}D_{n}$ bits of data executed on RSU1, the processing time is

(8) $$\begin{array}{c} t_{n}^{RSU1,ex}=\frac{C_{n}\alpha_{n}D_{n}}{f_{n}^{r1}}. \end{array}$$

where $f_{n}^{r1}$$\left(0\leq f_{n}^{r1}\leq F_{RSU1}\right)$ represents the computing resources allocated for task $T_{n}$ by RSU1 and $F_{RSU1}$ is the maximum computing capability of RSU1. Thus, the energy consumption of RSU1 is

(9) $$\begin{array}{c} E_{n}^{RSU1,ex}=p_{n}^{RSU1}t_{n}^{RSU1,ex}=k_{r1}C_{n}\alpha_{n}D_{n}{\left(f_{n}^{r1}\right)}^{2}, \end{array}$$

where $p_{n}^{RSU1}=k_{r1}{\left(f_{n}^{r1}\right)}^{3}$ is the power consumption of RSU1 and $k_{r1}$ is a chip coefficient related to the power available to RSU1 [3].

### 3.4. Problem Formalization

We have already obtained the delay and energy consumption for local computation and V2I offloading; however, these cannot simply be added together. Based on our scenario in Figure 1, vehicle *n* may be located in different positions, with concurrent differences in offloading delay and energy consumption.

#### 3.4.1. Delay Analysis

**Position 1:** If vehicle *n* is in the coverage of RSU1 (β=0) or RSU2 (α=0), the task can only be offloaded to one RSU or performed locally. Let $t^{off}$ be the offloading delay, which is $t^{off}=t^{RSU1,up}+t^{RSU1,ex}$ or $t^{off}=t^{RSU2,up}+t^{RSU2,ex}$.

**Figure 2:**
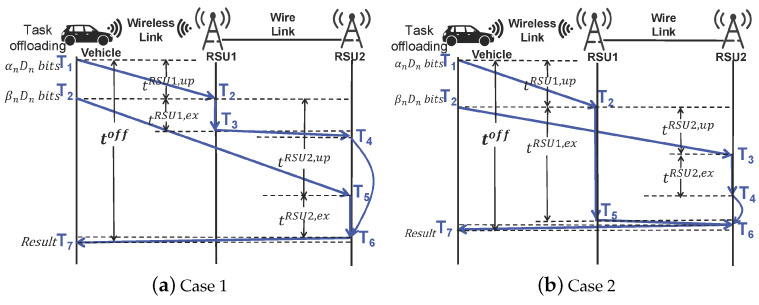
Delay analysis of two offloading strategies.

**Position 2:** If vehicle *n* is within the cross-coverage area of adjacent RSUs, it may drive out of RSU1 during offloading, causing service interruption. To solve this issue, the two RSUs can collaborate with each other and make optimal offloading decisions. There is a period of overlapping time between uploading to RSU2 $t^{RSU2,up}$ and processing on RSU1 $t^{RSU1,ex}$. For example, after $\alpha_{n}D_{n}$ bits of data are transferred to RSU1, the vehicle can connect to RSU2 to upload $\beta_{n}D_{n}$ bits of data while RSU1 is computing. To this end, we consider two task offloading cases with different offloading delays according to the execution time of the $\alpha_{n}D_{n}$-bit data on RSU1, as shown in Figure 2. We ignore the resulting transmission delay between RSUs with wired connections [14]. The blue arrow illustrates the critical path for processing data on RSU1 and RSU2.

Case 1: In Figure 2a, vehicle *n* with a data size of $\alpha_{n}D_{n}$ bits is first transmitted to the RSU1 server ($T_{2}$-$T_{1}$). The execution time of $\alpha_{n}D_{n}$ bits of data on RSU1 is less than the time to transmit $\beta_{n}D_{n}$ bits of data to RSU2 ($t^{RSU1,ex}$ < $t^{RSU2,up}$). When RSU1 finishes ($T_{3}$), the results are transmitted to RSU2 ($T_{4}$-$T_{3}$). Afterwards, RSU2 processes the $\beta_{n}D_{n}$ bits of data ($T_{6}$-$T_{5}$) and then returns the calculations of RSU1 and RSU2 to the vehicle *n* ($T_{7}$-$T_{6}$). Let $t^{off}$ ($T_{7}$-$T_{1}$) be the offloading delay, which includes the delay of uploading to RSU1 $t^{RSU1,up}$, the delay of uploading to RSU2 $t^{RSU2,up}$, and the delay of execution on RSU2 $t^{RSU2,ex}$, which can be expressed as

(10) $$t_{n}^{\mathrm{off}\;}=t_{n}^{RSU1,up}+t_{n}^{RSU2,up}+t_{n}^{RSU2,ex}.$$

Case 2: Unlike Case 1, in Figure 2b the execution time of $\alpha_{n}D_{n}$ bits of data on RSU1 is more than the time needed to transmit $\beta_{n}D_{n}$ bits of data to RSU2 ($t^{RSU1,ex}$ > $t^{RSU2,up}$). Therefore, after RSU2 completes the calculation task ($T_{4}$-$T_{3}$), it is necessary to receive the results of RSU1 ($T_{6}$-$T_{5}$) and return them to vehicle *n* ($T_{7}$-$T_{6}$). Let $t^{off}$ ($T_{7}$-$T_{1}$) be the offloading delay, which includes the delay of uploading to RSU1 $t^{RSU1,up}$ and the delay of execution on RSU1 $t^{RSU1,ex}$, which can be expressed as

(11) $$\begin{array}{c} t_{n}^{\mathrm{off}\;}=t_{n}^{RSU1,up}+t_{n}^{RSU1,ex}. \end{array}$$

Thus, based on the above two case analyses, the offloading delay of vehicle *n* can be expressed as

(12) $$\begin{array}{c} t_{n}^{\mathrm{off}\;}=t_{n}^{RSU1,up}+\max\left\{t_{n}^{RSU1,ex},t_{n}^{RSU2,up}+t_{n}^{RSU2,ex}\right\}. \end{array}$$

#### 3.4.2. Energy Analysis

The offloading energy consumption of vehicle *n* is independent of location, including transmission and calculation in RSUs. Let $E_{n}^{off}$ be the offloading energy consumption of vehicle *n*, which is expressed as

(13) $$\begin{array}{c} E_{n}^{\mathrm{off}\;}=E_{n}^{RSU1,up}+E_{n}^{RSU1,ex}+E_{n}^{RSU2,up}+E_{n}^{RSU2,ex}. \end{array}$$

#### 3.4.3. Task Offloading Problem

Our goal is to minimize the delay and energy consumption of all vehicles in order to arrive at a reasonable resource allocation strategy. Hence, the offloading ratio, communication resource, and computing resource allocation all need to be optimized. Here, we use *X* to represent the offloading ratio, *Y* to represent the allocation (local, RSU1, and RSU2) of computing resources, and *Z* to represent the uplink subchannel allocation of vehicle *n* to RSU1 and RSU2 over subchannel *m* and *n*; *X*, *Y*, and *Z* can respectively be denoted by

(14) $$\begin{array}{cc} \; & X=\left\{\alpha_{1},\alpha_{2},\ldots ,\alpha_{N},\beta_{1},\beta_{2},\ldots ,\beta_{N},\gamma_{1},\gamma_{2},\ldots ,\gamma_{N}\right\},\\ \; & Y=\left\{f_{1}^{l},f_{2}^{l},\ldots ,f_{N}^{l},f_{1}^{r1},f_{2}^{r1},\ldots ,f_{N}^{r1},f_{1}^{r2},f_{2}^{r2},\ldots ,f_{N}^{r2}\right\},\\ \; & Z=\left\{a_{1}^{1},\ldots ,a_{1}^{M},\ldots ,a_{N}^{1},\ldots ,a_{N}^{M},b_{1}^{1},\ldots ,b_{1}^{K},\ldots ,b_{N}^{1},\ldots ,b_{N}^{K}\right\}. \end{array}$$

Therefore, the multi-objective optimization problem can be expressed as

(15) $$\begin{array}{cc} \; & \min_{\left\{X,Y,Z\right\}}t=\sum_{n=1}^{N}\max\left\{t_{n}^{l},t_{n}^{\mathrm{off}\;}\right\},\\ \; & \min_{\left\{X,Y,Z\right\}}E=\sum_{n=1}^{N}\left(E_{n}^{l}+E_{n}^{\mathrm{off}\;}\right). \end{array}$$

Accordingly, the problem in Formula (15) can be rewritten under constraints as Formula (16).   

(16) $$\begin{array}{cc} \min_{\left\{X,Y,Z\right\}}t & =\sum_{n=1}^{N}\max\left\{\frac{C_{n}\gamma_{n}D_{n}}{f_{n}^{l}},\frac{\alpha_{n}D_{n}}{R_{n}^{RSU1,up}}+\right.\left.\max\left\{\frac{C_{n}\alpha_{n}D_{n}}{f_{n}^{r1}},\frac{\beta_{n}D_{n}}{R_{n}^{RSU2,up}}+\frac{C_{n}\beta_{n}D_{n}}{f_{n}^{r2}}\right\}\right\}\\ \min_{\left\{X,Y,Z\right\}}E= & \sum_{n=1}^{N}\left\{k_{n}C_{n}\gamma_{n}D_{n}{\left(f_{n}^{l}\right)}^{2}+\sum_{m=1}^{M}p_{n,m}^{RSU1,up}t_{n}^{RSU1,up}\right.+k_{r1}C_{n}\alpha_{n}D_{n}{\left(f_{n}^{r1}\right)}^{2}\\ \; & +\sum_{k=1}^{K}p_{n,k}^{RSU2,up}t_{n}^{RSU2,up}\left.+k_{r2}C_{n}\beta_{n}D_{n}{\left(f_{n}^{r2}\right)}^{2}\right\} \end{array}$$

$$\begin{array}{c} \begin{array}{ccc} \;\;\;\;\;\;\;\;\;\;\;\;\;\;\;\;\;\;\;\;\;\;\;\;\;\;\;\;\;\;\;\;\;\;\;\;\;\;\;\;\;\;s.t. & 0\leq \alpha_{n},\beta_{n},\gamma_{n}\leq 1,\;n\in N & \left(16a\right)\\ \; & \alpha_{n}+\beta_{n}+\gamma_{n}=1,\;n\in N & \left(16b\right)\\ \; & 0\leq f_{n}^{l}\leq F_{n},\;n\in N & \;\;\;\;\;\;\;\;\;\;\;\;\;\;\;\;\;\;\;\;(16c)\\ \; & 0\leq f_{n}^{r1}\leq F_{RSU1},\;0\leq f_{n}^{r2}\leq F_{RSU2},n\in N & \left(16d\right)\\ \; & \sum_{n=1}^{N}f_{n}^{r1}\leq F_{RSU1},\;\sum_{n=1}^{N}f_{n}^{r2}\leq F_{RSU2} & \left(16e\right)\\ \; & \;a_{n}^{m},\;b_{n}^{k}\in \left\{0,1\right\},\;n\in N,m\in M,k\in K & \left(16f\right)\\ \; & \sum_{n=1}^{N}a_{n}^{m}\leq 2,\;\sum_{n=1}^{N}b_{n}^{k}\leq 2 & \left(16g\right)\\ \; & t_{n}^{RSU1,up}\;\leq \;t_{stay},\;n\in N & \left(16h\right)\\ \; & t_{n}\leq \;t_{max},\;\;n\in N & \left(16i\right) \end{array} \end{array}$$

The constraints in (16a) and (16b) show the relationships between α, β, and γ; (16c), (16d), and (16e) are the computing capacity constraints for vehicle *n* and the RSUs; and (16f) shows uplink communication resource allocation as a binary vector. In (16g),a subchannel is limited to being allocated to two vehicles at most, as SIC makes the network more complex; (16h) bounds the transmission latency of vehicle *n* to the RSU to within the current RSUs, where the dwell time within the coverage of the current RSU is $t_{stay}$; (16i) limits the delay; and (16h) and (16i) are related to the current vehicle speed.

## 4. Computation Offloading Algorithm

Formula (16) contains continuous and binary variables; thus, this problem is a Mixed-Integer Nonlinear Programming (MINLP) problem, which is NP-hard [11]. Such problems are difficult to solve using traditional optimization methods, such as game theory [5], convex optimization [5], etc., because they are more suitable for problems with low complexity and a single objective [9]. Therefore, we propose a multi-objective computing offloading method for VEC (MOV) considering individual QoS requirements, with an improved NSGA-II algorithm used to generate the Pareto-optimal solutions [15]. Below, we provide a detailed introduction to our method.

**Figure 3:**
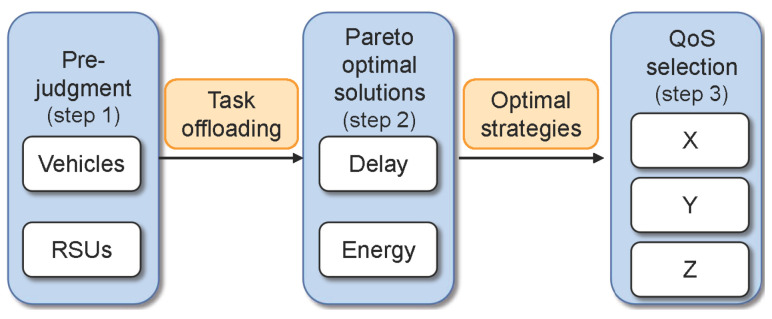
MOV overflow.

The NSGA-II algorithm is based on biological evolution, and is suitable for complex and multi-objective optimization problems [15]. Compared with the original NSGA algorithm, it achieves faster and more accurate search performance, and is widely used. There are three main improvements: (1) a fast non-dominated sorting algorithm; (2) congestion degree comparison; and (3) an elite strategy. Based on NSGA-II, we propose using MOV to solve the offloading optimization problem by satisfying the application-specific QoS. The novelty is that the vehicle can selectively offload data according to different priorities of tasks and tolerance delays related to vehicle speed, allowing it to accelerate execution based on the previous optimal solution. An overview of our algorithm is shown in Figure 3. In Step 1, the vehicle judges whether execution should occur locally or be offloaded to RSUs for help according to the priority of the task. In Step 2, an improved NSGA-II is used to find the Pareto-optimal solutions minimizing delay and energy consumption for the tasks to be offloaded, where we design a special coding scheme based on the VEC scene. Our method can accelerate the algorithm execution speed based on the previous optimal solutions. In Step 3, a QoS model is established to carry out the optimal offloading strategy by achieving the individual QoS requirements of different applications. The MOV algorithm is shown in Algorithm 1.

**Algorithm 1** Multi-Objective computing offloading algorithm for VEC, MOV
**Input:** The number of vehicles *N*, the offloading task $T_{n}(n\in N)$, the NSGA-II algorithm parameters $S,K,P_{c},P_{m}$
**Output:** The optimal offloading strategy of vehicles, [X,Y,Z].1:Separate task categories according to vehicle pre-judgment2:Initialize the population P(gen) according to a priori knowledge3:Encode the variables ${\left[X,Y,Z\right]}_{d}$ to each chromosome $C_{i}$, i∈S4:Some chromosomes were selected to produce offspring O(gen)5:**for**gen in *K* **do**6:    PO(gen)←P(gen)∪O(gen)7:    Calculate the fitness values $t_{i}$ and $E_{i}$ of each chromosome $C_{i}$, i∈2×S8:    PO(gen)← Non-dominated sorting(PO(gen))9:    PO(gen)← Crowding distance(PO(gen))10:    P(gen+1)← Elite strategy selects(PO(gen))11:    O(gen+1)← Crosses and mutates(P(gen+1))12:    gen←gen+113:Get the Pareto-optimal solution set Z according to the limits in (16)14:Store $\left\{{\left[X,Y,Z\right]}_{z}\right\}$ and $\left\{{\left[t,E\right]}_{z}\right\}$ for Z, z∈Z15:**for***z* in *Z* **do**16:    $QoS^{z}$←$QoSmodel(\lambda_{n},\mu_{n})$, n∈N17:optimal_z ←$max(QoS^{z})$18:**return** optimal_z


### 4.1. Step 1: Vehicle Prejudgment

In actual traffic scenarios, the scale of the joint optimization problem increases rapidly with an increasing number of vehicles. If all tasks are optimized in a centralized way, this results in severe complexity. Thus, we consider that different tasks have different priorities in terms of delay to offload partial data to RSUs. We divide tasks into two categories, high-priority applications (HPA) and low-priority applications (LPA), according to their different delay priorities. An HPA is a task with a high delay requirement, such as one related to autonomous driving or road safety. The on-board system of the vehicle should always be designed to have sufficient capacity to meet the resource needs of such tasks [16]. Thus, for this type of task, we consider processing it locally. For LPAs, tasks with relatively low latency requirements, such as navigation and entertainment activities, can be offloaded to the RSUs servers for help. At the same time, we consider that the maximum tolerance delay of the LPA is related to the current vehicle speed, which means that a vehicle traveling at a low speed can tolerate a relatively high delay compared to a vehicle at high speed. Here, we use a function to describe the maximum delay tolerance model according to vehicle speed for an LPA as follows [17]:

(17) $$\begin{array}{cc} t_{\max}\left(s_{n}\right) & =T_{LPA}\frac{1}{\sqrt{2\pi }\rho }\exp\left(-\frac{s_{n}^{2}}{2\rho^{2}}\right)/\left(\frac{1}{\sqrt{2\pi }\rho }\exp\left(-\frac{s_{\max{\;}^{2}}}{2\rho^{2}}\right)\right)\\ \; & =\exp\left(-\frac{s_{n}^{2}-s_{\max}^{2}}{2\rho^{2}}\right)T_{LPA}. \end{array}$$

where $T_{LPA}$ is the delay threshold for the LPA, which is consistent. To ensure that the probability of vehicle speed data within the allowed maximum vehicle speed is within the 95 percent confidence interval, we denote the standard deviation as ρ, expressed as ρ = $s_{max}/1.96$ [18], where $s_{n}$ and $s_{max}$ represent the current and maximum vehicle speeds. This solves the time limitation of delay-sensitive tasks while reducing resource competition among multiple vehicles according to vehicle prejudgment of task types and vehicle velocity.

### 4.2. Step 2: Obtaining the Pareto-Optimal Solutions

(1) Encoding. Here, we combine the NSGA-II algorithm into the actual vehicle offloading problem and design a real coding scheme based on the offloading ratio and resource allocation which contains vectors and matrices. In this algorithm, each chromosome $C_{i}$ in the population represents an offloading strategy for the computation tasks collection *N*, where each gene represents an offloading decision variable of vehicle *n*. A set of chromosomes/solutions form a population. For example (see line 2 of Algorithm 1), a chromosome $C_{i}$ denotes a set of *X*, *Y*, and *Z*, which can be defined as an array $C_{i}={\left[X,Y,Z\right]}_{d}$, where *d* is the size of the array and is defined as d=5×N+2×2×M. In each $C_{i}$, the size of *X* and *Y* is 2×N and 3×N in (14), respectively, which are vectors. The size of *Z* is 2×2×M, which indicates that a subchannel can be allocated to two vehicles at most in the uplink of RSU1 and RSU2. Further, it can be extended into a 0–1 matrix with size N×M to deal with the subchannel allocation problem.

(2) Fitness function and constraints. The fitness functions in this paper include two categories, namely, delay and energy consumption, as presented in (15). They both must be minimized, and we aim to achieve trade-offs among the two objectives while satisfying the edge computing capability, delay tolerance, and task assignment constraints in (16a)–(16i).

(3) Initialization. In this algorithm, certain parameters should be initialized, including the population size *S*, maximum iteration *K*, crossover probability $p_{c}$, and mutation probability $p_{m}$. In a chromosome $C_{i}$, the variable corresponding to each gene is initialized with the constraints ranges in (16a), (16c), (16d), and (16f). Under the generation rule of a single chromosome, multiple chromosomes can be randomly generated to form an initial population $P=\{C_{1},C_{2},\cdots ,C_{S}\}$, where each chromosome $C_{i}$ contains two fitness values, which can be defined as $C_{i}=\left\{t_{i},E_{i}\right\}$. In Algorithm 1 lines 2–3, the novelty lies in initialization with the stored offloading decision variables, which can greatly reduce the evolutionary process.

(4) Selection. In parent population P(gen), we select the chromosomes with the best fitness as parents to produce offspring O(gen) by performing tournament selection, as this method has a low computational cost. Then, the parent population P(gen) and the offspring population O(gen) with size *S* form a new population PO(gen) with size 2S. Next, we compute the fitness value of all chromosomes in PO(gen) and obtain the non-dominated rank and crowding distance of each chromosome. Finally, *S* chromosomes are selected to form the next generation parent P(gen+1) based on the elite strategy, which has better performance (see lines 4–10).

(5) Crossover and mutation. After generating the parent population P(gen+1) with size *S*, we recombine the offloading variables of different chromosomes for crossover operation to generate new solutions. Certain variables are modified according to the mutation probability, thereby avoiding local convergence (see line 11).

(6) Iteration. Based on crossover and mutation for P(gen+1), let gen=gen+1 and return to the previous steps (4)–(5) until the stop number of iterations is reached (in line 12).

(7) Storage. After all iterations, the Pareto-optimal solutions are saved as a priori knowledge, as NSGA-II is a one-shot planning algorithm which tries to compute the optimal offloading strategy according to the current system state and may have high complexity. Using this prior knowledge-based mechanism, the decision variables are directly used for encoding in the next optimization, making the optimal strategy faster and reducing time complexity (see line 13–14).

### 4.3. Step 3: Selection of Optimal Offloading Strategy

After obtaining the optimal Pareto solution set *Z*, an offloading strategy *z* represents the hybrid offloading decisions of *N* tasks that meet the minimum delay and energy cost. Note that our goal is to provide a flexible QoS model to satisfy different performance metrics. Thus, we propose a concrete objective with QoS. Then, the optimal offloading strategy that maximizes the QoS value of all vehicles is selected.

As mentioned above, $\lambda_{n}$ and $\mu_{n}$ denote the weight of vehicle *n* corresponding to delay and energy consumption, and their sum is equal to 1. The values of the delay and energy consumption variables are negative indicators, which mean that lower delay and energy consumption values represent a better solution. A relatively small $\lambda_{n}$ indicates relatively high tolerance with respect to latency, while a larger value indicates that the application is deeply concerned with real-time performance. We define the QoS model of vehicle *n* with strategy *z* as follows:

(18) $$QoS_{z}^{n}=\frac{\lambda_{n}\left(t_{z}^{\max}-t_{z}^{n}\right)}{t_{z}^{\max}-t_{z}^{\min}}+\frac{\mu_{n}\left(E_{z}^{\max}-E_{z}^{n}\right)}{E_{z}^{\max}-E_{z}^{\min}}.$$

where $t_{z}^{max}$ and $E_{z}^{max}$ are the maximal latency and maximal energy consumption with strategy *z*. The goal of the MOV is to make the optimal offloading decisions for QoS maximization. The MOV takes as inputs the sum of the QoS values of all vehicles with each solution, then outputs the optimal offloading strategy with the largest QoS value.

*Complexity analysis.* Finally, we discuss the complexity of MOV. Using prior knowledge, the complexity of initializing the population of the size *S* is O(1). Compared with random reinitialization, our MOV is reduced by $O(S\times N^{\prime }\times d)$, where $N^{\prime }$ is the number of tasks to be offloaded with low delay priority and *d* is the dimension of decision variables mentioned above. To this end, the time complexity of our method using the MOV is $O(S^{2})$. Based on experiments, after vehicle prejudgment in Step 1, the average running time of our algorithm is less than 0.1 s. In addition, the memory space occupied by the storage of a priori knowledge is 5 KB, which is acceptable.

## 5. Evaluation

In this section, we evaluate our proposed method MOV and compare it with the following schemes:

(1) Process-Local-Only (PLO): In this scheme, the tasks of all vehicles are processed locally.

(2) Offload-RSUs-Only (ORO): In this scheme, the tasks of all vehicles are offloaded to two RSUs for processing.

(3) PSOCO [3]: A state-of-the-art VEC offloading scheme which considers offloading the tasks to the nearest RSU, ignoring the possibility of collaboration between RSUs.

### 5.1. Simulation Setup

**Figure 4:**
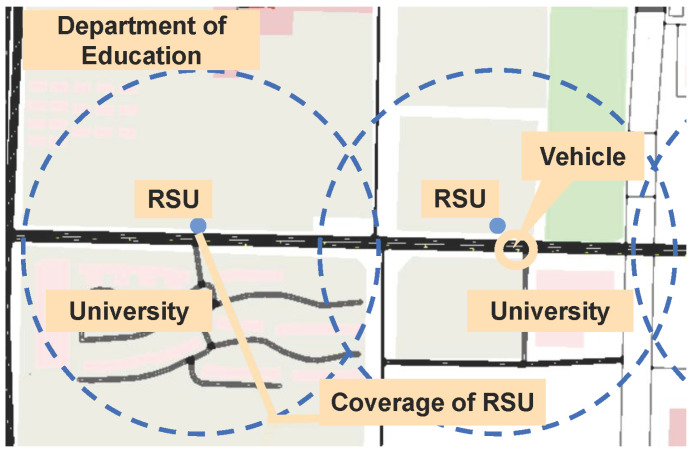
Evaluation scenario based on SUMO.

In this section, we our experiments based on realistic and simulated vehicle trajectories. Considering a realistic scenario of the Yanta area in Xi’an, China, we verify our experiments based on the GAIA Open Dataset containing mobility traces from DiDi Express [19]. We select a one-way two-lane road with a length of 1000 m. The dataset includes GPS data for Xi’an city collected over a 30 day time range from 1 November 2016 to 30 November 2016. Each trajectory consists of a vehicle ID, timestamp, longitude, and latitude. After preprocessing the raw data, the vehicle trajectories of 50–90 vehicles are extracted. We take the average value from ten experiments as the experimental result. Additionally, we carry out simulation experiments with different traffic congestion levels. We use OpenStreetMap [20] to import the Xi’an map into SUMO and generate vehicle trajectory data, as shown in Figure 4. The two RSUs are located at the roadside and their coverage is overlapped. There are *N* vehicles with different speeds on the road. The simulation parameters with RSU1 are shown in Table 1, and RSU2 is similar. For the simulation environment, we use a GPU-based server with an Intel Core i5-9400F CPU with 16 GB memory. The software environment is Python 3.7 on Ubuntu18.04 LTS.

### 5.2. Simulation Results

#### 5.2.1. Pareto-Optimal Solutions

To comprehensively examine the proposed MOV with vehicle prejudgment and speed-aware delay constraint, the results of our method are compared with that of ORO, PLO, and PSOCO. The different performances of these algorithms when the number of vehicles is 50, 60, and 70, respectively, are shown in Table 3. To be specific, it concludes the min/max delay and the min/max energy consumption of the Pareto-optimal solution. At the same time, we compare the QoS values of the different algorithms, in which each task has different performance indicators. The results presented here are from ten experiments. In Table 3, it can be seen that ORO has the shortest delay and the highest energy consumption compared to the other algorithms, while PLO is the opposite, with the highest delay and the lowest energy consumption. As for QoS value, ORO and PLO have the lowest results with different numbers of vehicles. Because they only consider one offloading destination, the limited computing capacity of the vehicle and the cost of excessive communication resources are ignored. In the same circumstances, MOV has a better result than PSOCO in both delay and energy consumption. In terms of QoS value, our method shows the best results with different numbers of vehicles. This is because our proposed algorithm can better consider the differences between applications with different performance indicators, especially for HPA, allowing the offloading and resource allocation strategy to be adjusted for each vehicle and thereby further reducing task completion delay and energy consumption. We consider the maximum tolerance delay related to the current speed as well.

**Figure 5:**
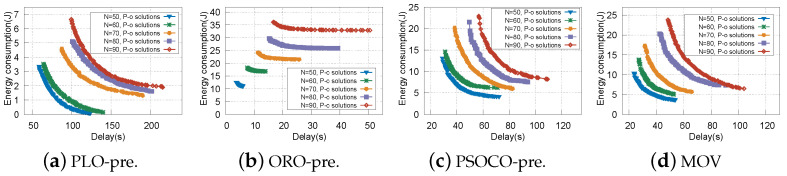
Pareto-optimal solutions under different schemes.

In order to fairly and specifically compare the delay and energy consumption, we establish five sets of experiments with vehicle prejudgment based on the above four offloading schemes, with the number of vehicles between 50–90. Realistic and simulated vehicle trajectories are used at different speeds. The Pareto-optional solutions with different schemes are shown in Figure 5. It can be easily observed that the delay and energy consumption of all tasks increases for any scheme as the number of vehicles increases. This is because more vehicles have more tasks to process, resulting in more delays and energy consumption given the limited RSU resources. In addition, a trade-off between two objectives is obtained for MOV and PSOCO-pre (the PSOCO algorithm with vehicle prejudgment), which can guide optimal offloading decisions made for different types of tasks. If a solution with lower delay is selected, this produces higher energy consumption. This is because more vehicles have high requirements for delay, which leads to more tasks being offloaded to RSUs to reduce the delay due to the powerful computing capability of the RSUs. However, this may increase energy consumption because of the occupation of edge resources. For example, when the number of vehicles *N* is 70, in Figure 5d, among the “N=70 P-o solutions” there is a solution with the delay of about 35 s and energy consumption of about 13 J. This means that more vehicles results in high requirements for delay, while few vehicles leads to high requirements for energy consumption, which in turn leads to more tasks being offloaded to RSUs for processing to reduce the overall delay.

**Figure 6:**
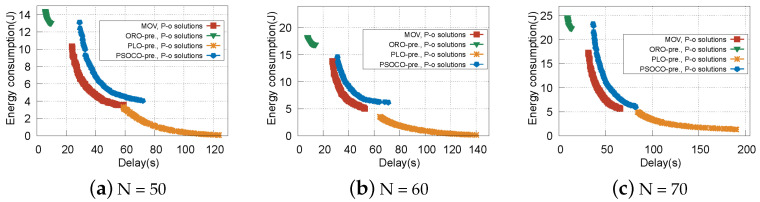
Pareto-optimal solutions of MOV, ORO-pre, PLO-pre, and PSOCO-pre with different numbers of vehicles.

Next, we clearly describe Pareto-optimal solutions of the four schemes with different numbers of vehicles, as shown in Figure 6. It can be seen that ORO-pre (the ORO algorithm with vehicle prejudgment) has a higher energy consumption and a lower delay, while PLO-pre (the PLO algorithm with vehicle prejudgment) has a higher delay and a lower energy consumption. The reason for this is that if all tasks are offloaded, more RSUs resources are allocated to transmit and process tasks, which increases energy consumption due to resource utilization. However, if all tasks are processed locally, the delay is greatly increased. This is because vehicle computation capacity is limited to processing tasks. As for PSOCO-pre and MOV, the two indexes are between those of ORO-pre and PLO-pre, and our proposed MOV has better performance than PSOCO-pre. This is because we specifically consider the offloading decision-making at the coverage boundary of the two RSUs, in which RSE cooperates to complete tasks that avoid service interruption and the imbalance of RSU load. However, for PSOCO-pre, only the nearest RSU is mentioned, in order to prevent the vehicle driving out the RSU during task transmission, meaning that more data is processed locally, resulting in more delay.

#### 5.2.2. The Validity of the Proposed Strategy

**Figure 7:**
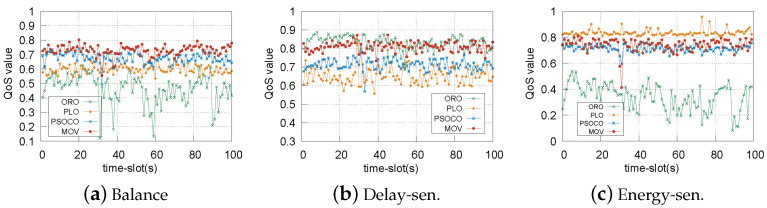
QoS values of ORO, PLO, PSOCO, and MOV with different requirements.

The unstable communication condition of each time slot is a key challenge during the task offloading. Figure 7 shows the performance comparison of the QoS value on 100 slots with unstable link bandwidths. Our MOV algorithm achieves better performance compared with other algorithms under three average QoS weights : “Balanced”(λ = 0.5, μ = 0.5), “Delay-sensitive”(λ = 0.8, μ = 0.2), and “Energy-sensitive”(λ = 0.2, μ = 0.8). It can be seen that: (1) MOV achieves a consistently high QoS value and the highest QoS for “Balance”, as it can use a better offloading strategy considering different task requirements through cooperation and the real-time speeds related to tolerance delay. PSOCO shows a smaller QoS value than MOV, as it only considers the nearest RSU to offload tasks without cooperation of RSUs, resulting in a suboptimal strategy. For PLO and ORO, the QoS values are the lowest because the cooperation between vehicles and edges is ignored. (2) In Figure 7b,c, compared to the ORO, PLO, and PSOCO algorithms, the MOV algorithm shows better QoS performance. Although ORO achieves the highest QoS value for “Delay-sen”, its QoS value is the lowest, and it has a large fluctuation for “Energy-sen”. This is because, while ORO achieves the lowest delay, as mentioned above, because is very suitable for delay-sensitive tasks, for energy-sensitive tasks its QoS value is the lowest and fluctuates due to unstable network conditions. On the contrary, while PLO is suitable for “Energy-sen”, it is the worst for “Delay-sen”. This is because PLO achieves the lowest energy consumption, which results in the highest delay. Moreover, our algorithm MOV achieves the highest QoS value compared to PSOCO, especially for “Delay-sen”, because it considers the speed-related tolerance delay limit, which further reduces task completion delay. Therefore, MOV is always a better choice to satisfy the QoS requirements of any task.

**Figure 8:**
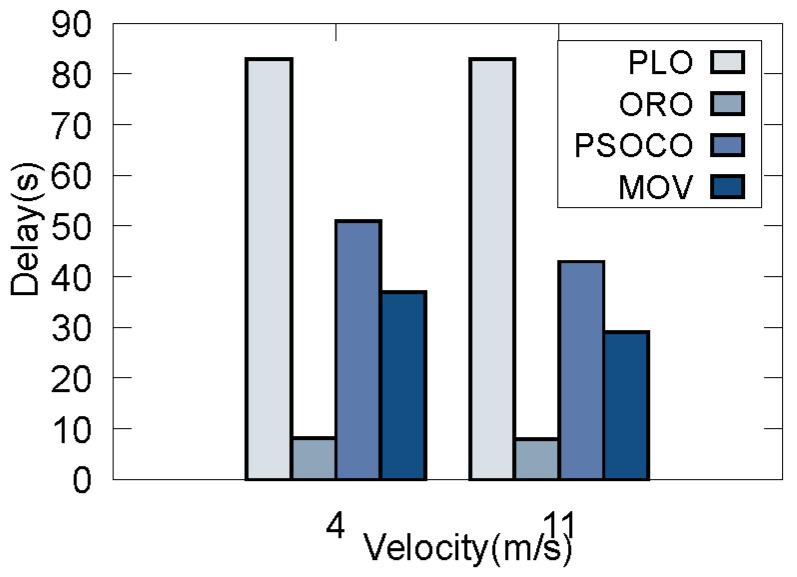
Results of different velocities on delay.

**Figure 9:**
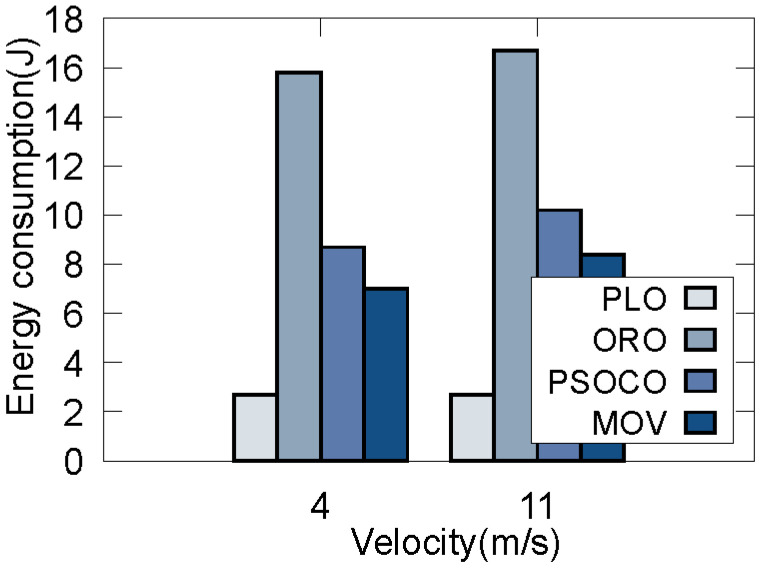
Results of different velocities on energy cost.

Considering that there may be traffic congestion on a real road, in this scenario the speed of the vehicles is low. Thus, when the number of vehicles with different demands at a certain time is 60, we compare the performance indicators of different traffic congestion conditions, with the average speed of all vehicles being 4 m/s and 11 m/s based on simulated data in Figure 8 and Figure 9. It can be seen that: (1) for MOV, there is a higher delay and lower energy consumption when the average speed is 4 m/s compared to when it is 11 m/s. The reason for this is that a higher velocity causes more offloading failures, and the vehicle drives out of the current RSU during offloading. Therefore, the vehicle tends to offload the task to RSUs with low delay when the vehicle speed is high, which leads to greater energy consumption during to task processing. (2) Compared with PSOCO, our method achieves lower delay and energy consumption at any speed, as PSOCO ignores task offloading failure due to the high mobility of the vehicle. As our method considers vehicle prejudgment in Step 1, each vehicle can make the optimal offloading decision. (3) While ORO achieves the lowest latency, it produces the largest energy consumption. On the contrary, while PLO achieves the lowest energy consumption, its delay is too high, independent of vehicle speed. As a result, our algorithm can achieve optimal performance, and is able to select the maximum QoS value relative to the speed of the vehicle.

**Figure 10:**
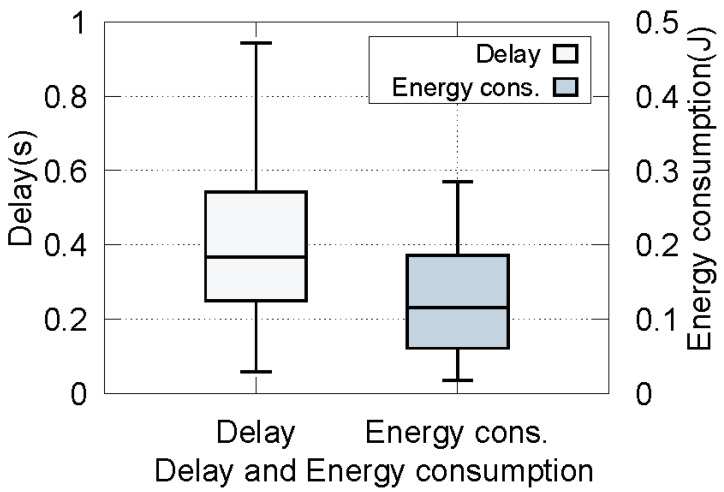
Delay and energy consumption of all vehicles.

In order to show the performance of our MOV, an optimal offloading strategy is selected under the QoS model; in this scenario, the number of vehicles is 50. The delay and energy consumption of each vehicle are shown in Figure 10 with box plots. In Figure 10, most tasks can be finished in about 0.38 s and 0.12 J within maximum tolerance delay. This is because our method comprehensively considers cooperation between the RSUs and limits the possibility of task execution failure, which ensures the delay requirements for delay-sensitive tasks and avoids RSU overload or resource waste.

Furthermore, we compare MOV with the state-of-the-art existing technique P-PPO [21], as shown in Table 4. P-PPO is a recent study that adopts deep reinforcement learning (DRL) to reduce delay and energy consumption with five vehicles. Here, we use the Proximal Policy Optimization (PPO) algorithm [22] to train a neural network model to make the optimal offloading strategy, using the RSU as an agent. We compare the reward value under three average QoS weights: “Balanced”, “Delay-sensitive”, and “Energy-sensitive”, with the number of vehicles set to 5, 7, and 10. If the number of vehicles is large, this leads to difficulties in model training and introduces problems with dimensionality due to the offloading strategy including the allocation of offloading ratio, communication resources, and computational resources. In Table 4, we set the reward value model as follows: reward=10/(10+λ×t+μ×e), where t and e are the delay and energy consumption, respectively, of each vehicle. In the P-PPO algorithm, the reward value model is used as the reward function. In the MOV algorithm, the result is converted into reward value according to the reward model after obtaining the optimal offloading decisions. From Table 4, it can be seen that the results of MOV with different numbers of vehicles are better than those of P-PPO. This is because our method can select the optimal solution according to the QoS model, unlike P-PPOm which obtains a solution each time using a neural network model. As the number of vehicles increases, resulting in the dimension of optimization variables increasing, it is difficult for the algorithms to find the optimal solution, and the reward value decreases, especially for P-PPO. At the same time, we find that the neural network model is very difficult to train when the number of vehicles is 50 because of the high dimensionality. Therefore, our proposed method has better performance than this state-of-the-art existing technique.

Finally, in Table 5, we compare the performance of the improved MOV with the previous work MOV-Simple [23], with the number of vehicles set to 50. Our improved MOV algorithm is significantly better than the previous MOV-S algorithm in terms of OoS value, which means that MOV can make more optimal offloading decisions for tasks with different requirements. This is because we further divide tasks into two categories to reduce the completion time according to the delay priority. Moreover, it can be seen that the task completion delay decreases significantly, while the energy consumption increases only a little, because more tasks are offloaded in order to reduce latency. This is suitable for a Pareto-optimal solution, and the QoS value is improved. In conclusion, our improved multi-objective computing offloading method for VEC (MOV) can improve QoS value and be applied in multiple applications.

## 6. Related Work

**V2V-based VEC offloading:** In VEC, many works offload computation tasks to powerful vehicles for processing. Zhang et al. [1] proposed putting underutilized V2V link resources to use in order to help with task offloading. Chen et al. [24] focused on the allocation of computing resources and developed a task-offloading framework V2V to gain a shorter task execution time. Lin et al. [25] proposed a predicted k-hop-limited multi-RSU-considered (PKMR) vehicle-to-vehicle-to-roadside unit (VVR) data offloading method inside a multi-access edge computing (MEC) server, which is able to consider the time-extended prediction mechanism to find the potential VVR paths and network conditions. However, the vehicle computation capacity is dynamic and the V2V link is unstable, which makes it difficult to choose a proper vehicle to offload. Unlike the above works, we consider an offloading strategy based on V2I for better reliability.

**V2I-based VEC offloading:** Recently, a large number of works have proposed V2I-based VEC offloading optimization problems. Ning et al. [26] proposed an offloading scheme that considers task offloading and content caching delay. Ning et al. [13] tried to maximize the achievable transmission rate by integrating cellular and RSU approaches, taking advantage of NOMA and MEC technologies. Zhou et al. [14] optimized energy consumption and formulated an energy-efficient workload offloading problem with explicit consideration of the overall energy consumption and latency. However, they focused on only one performance index. Wan et al. [8] formulated a multi-objective optimization problem to select suitable destination ENs, with the aims of minimizing the offloading delay and cost and realizing the load balance of the ENs. However, they ignored the effects of time-varying networks and rational resource allocation in real scenarios. Different from the above works, we jointly consider task scheduling, communication resources, and computing resources allocation according to different QoS requirements in order to minimize both delay and energy consumption in a vehicular network.

To solve this problem, RL-based methods are often adopted [7,27,28]. Dai et al. [7] developed an asynchronous task offloading algorithm inspired by the ideas of asynchronous advantage actor-critic (A3C) and deep Q-networks (DQN), which achieves fast convergence. Zheng et al. [27] proposed a digital twin-empowered task-offloading problem for IoV and developed a DRL-based framework to handle huge state spaces; their approach exploits an asynchronous advantage actor-critic algorithm to accelerate neural network training. Elham Karimi et al. [28] formulated a new resource allocation problem to guarantee the required response time and utilized deep reinforcement learning to capture an optimal solution. Although the RL (DQN, DDPG) algorithm is comprehensive in dealing with the dynamic problem of edge resources, it is a black-box process and requires enough data to ensure its performance. Furthermore, it is difficult to train a powerful neural network model for use with real-time changes in the number of vehicles, particularly for large-scale cars, due to the curse-of-dimensionality problem. In our scene, variables that are coupled lead to difficulties in train. Unlike the above works, our method can flexibly reach the optimal offloading strategy with low complexity and for a large number of vehicles.

**Collaborative computing offloading:** The efficient usage of MEC servers is another key challenge. Zhang et al. [29] designed a two-layer offloading framework based on the multi-part offloading mode and the collaborations among small cell base station (SBS) servers to achieve the optimal user experience. However, this is not suitable for high-speed vehicles. For high vehicle mobility, Pang et al. [30] discussed task retransmission due to service handover, which increases the delay. Zhang et al. [1] designed a predictive combination-mode offloading scheme with the cooperation of vehicles. However, it cannot provide stable service due to time-varying topology. In addition, service migration technology is widely used. Yuan et al. [31] investigated the joint service migration and mobility optimization problem to meet delay requirements. However, frequent service interruptions may increase service costs, and it is necessary to make accurate location predictions, which is difficult. Unlike the above works, we propose an offloading framework without trajectory prediction and collaborative vehicle selection by relying on coordinating RSUs and finding the optimal offloading strategy.

## 7. Conclusions

In this paper, we consider a practical urban scenario in with overlapping coverage of RSUs. We present FEVEC, a Fast and Energy-efficient VEC framework, to make optimal offloading decisions that minimize delay and energy consumption based on collaboration between RSUs. Then, we formulate it as an MINLP problem to be solved. We introduce a multi-objective computing offloading method for VEC named MOV, which employs an improved NSGA-II algorithm with an a priori knowledge-based mechanism, and introduce a QoS model to find the optimal offloading strategy with low complexity. Furthermore, we add constraints on the maximum tolerated delay for tasks related to vehicle speed. Finally, based on the vehicle’s trajectory as determined by SUMO and a realistic dataset, extensive experimental results show the superiority of our algorithm in both energy consumption and delay.

According to the current works, our future research will focus on following several directions. (1) Flexible decision-making frequency: FEVEC achieves high QoS performance by adopting a mechanism based on the time slot and finding the optimal strategy at a fixed frequency. In future work, it could be possible change the frequency of decision-making according to the actual network conditions. For example, when the network condition is poor, the frequency of decision-making could be reduced, then increased again with good network conditions. (2) Reliable task scheduling: FEVEC assumes that the task can be completed at any slot. However, due to the lack of communication and computation resources, tasks might not be completed in the current time slot, and a portion of the data may need to be processed in the next time slot. In future work, we will focus on a more reliable task-scheduling strategy to minimize delay and energy consumption. (3) Efficient offloading algorithm: FEVEC applies a heuristic algorithm, NSGA-II, to solve the MINLP problem, achieving good results. Although it has a high time complexity, we propose an a priori knowledge-based mechanism to solve it. In the future, this could be replaced with more efficient multi-objective optimization algorithms.

## Figures and Tables

**Table 1:** Simulation parameters.

Descriptions	Parameter	Value
Coverage radius of RSU	r	200 m
The number of vehicles	*N*	50–90
Uplink bandwidth of RSU1	$W_{1}$	100 MHZ
The uplink transmission power of vehicle *n* with RSU1	$p_{n,m}^{RSU1,up}$	1 W
The uplink power gains of vehicle n with RSU1	$h_{n,m}^{RSU1,up}$	1
Path loss exponent	*v*	3.5
Coefficients related to power in vehicle and RSU1 [10]	$k_{n}$,$k_{r1}$	$1.25\times 10^{-26}$, $10^{-29}$
Local maximum processing capacity	$F_{n}$	$3\times 10^{9}$ cycles/s
RSU1 maximum processing capacity	$F_{RSU1}$	$1\times 10^{10}$ cycles/s
White Gaussian noise powers	σ	−100 dBm
The delay threshold for LPA	$T_{LPA}$	0.8 s
The speed of vehicle	*s*	0–60 km/h

**Table 2:** Key notations and descriptions.

Notations	Descriptions
*r*	A coverage radius of one RSU
*N*	The number of vehicles
$D_{n}$	The data size of the task on the vehicle *n*
$C_{n}$	Computation intensity (in CPU cycles per bit)
$t_{max}$	Delay tolerance of the task $T_{n}$
$d_{n}^{RSU}$	Distance between vehicle *n* and RSU
$\alpha_{n}$, $\beta_{n}$, $\gamma_{n}$	Offloading ratio of vehicle *n* to RSU1, RSU2 and local
$W_{1}$	Uplink bandwidth of RSU1
*M*	The number of subchannels in the uplink of RSU1
$p_{n,m}^{RSU1,up}$	The uplink transmission power of vehicle *n* to RSU1
$h_{n,m}^{RSU1,up}$	The uplink power gains of vehicle *n* to RSU1
$\sigma^{m}$	White Gaussian noise powers on subchannel *m*
*v*	Path loss exponent
$k_{n}$,$k_{r1}$	Coefficients related to power in vehicle *n* and RSU1
$f_{n}^{l}$,$F_{n}$	Processing capability for task $T_{n}$ and maximum processing capability of vehicle *n*
$f_{n}^{r1}$,$F_{RSU1}$	Processing capability for task $T_{n}$ and maximum processing capability of RSU1
$a_{n}^{m}$	Indicator indicating whether subchannel *m* is allocated to vehicle *n*

**Table 3:** The values of indicators using different algorithms obtained with different numbers of vehicles.

The Number of Vehicles	Algorithm	QoS Value	Delay (s)		Energy Consumption (J)	
			Min	Max	Min	Max
N=50	ORO	0.45	**6.811**	9.187	11.139	12.462
	PLO	0.47	58.858	123.820	**0.080**	3.332
	PSOCO	0.63	27.957	78.406	2.590	14.915
	MOV	**0.74**	23.834	53.703	3.180	10.342
N=60	ORO	0.52	**7.638**	14.002	16.807	18.243
	PLO	0.54	64.372	140.077	**0.147**	3.535
	PSOCO	0.65	39.507	88.564	3.261	16.070
	MOV	**0.71**	27.243	55.769	5.002	13.850
N=70	ORO	0.51	**8.439**	13.989	20.882	22.855
	PLO	0.49	83.505	190.345	**1.255**	4.950
	PSOCO	0.66	41.045	119.717	3.632	17.562
	MOV	**0.75**	31.626	65.433	5.669	17.310

Note: Bold indicates minimum delay and energy consumption.

**Table 4:** Reward values when using different algorithms with different numbers of vehicles.

The Number of Vehicles	Algorithm	Reward Value		
		Balance	Delay-sen.	Energy-sen.
N=5	P-PPO	0.94	0.93	0.94
	MOV	0.97	0.96	0.98
N=7	P-PPO	0.89	0.89	0.89
	MOV	0.92	0.94	0.90
N=10	P-PPO	0.87	0.83	0.87
	MOV	0.92	0.88	0.93

**Table 5:** Values of different indicators compared with MOV-S.

Algorithm	Delay (s)		Energy Consumption (J)		QoS Value		
	Min	Max	Min	Max	Balance	Delay-sen.	Energy-sen.
MOV-S. [23]	21.33	58.27	3.72	10.15	0.61	0.72	0.68
MOV	15.42	47.15	3.81	14.03	0.68	0.86	0.75

## Data Availability

Not applicable.
